# Supplementary material for: Sweat Proteomics in Cystic Fibrosis: Discovering Companion Biomarkers for Precision Medicine and Therapeutic Development
Source: Cells. 2022 Jul 31;11(15):2358. doi: 10.3390/cells11152358 (PMC9367602; doi:10.3390/cells11152358)
Supplement: Supplementary file 1 [file cells-11-02358-s001.zip › cells-1810510-supplementary.pdf]

Sweat proteomics in Cystic Fibrosis: discovering companion biomarkers for precision medicine and therapeutic development.

Bastien Burat[1a], Audrey Reynaerts[2], Dominique Baiwir[3], Maximilien Fléron[3], Sophie Gohy[4,5,6], Gauthier Eppe[1], Teresinha Leal[2b], Gabriel Mazzucchelli[1ab]

[1] Mass Spectrometry Laboratory, MolSys Research Unit, Liège Université, B-4000 Liège, Belgium; g.eppe@uliege.be (G.E.)

[2] Louvain Center for Toxicology and Applied Pharmacology (LTAP), Institut de Recherche Expérimentale et Clinique (IREC), Université Catholique de Louvain, B-1200, Brussels, Belgium; audrey.reynaerts@saintluc.uclouvain.be (A.R.); teresinha.leal@uclouvain.be (T.L.)

[3] GIGA Proteomics Facility, Liège Université, B-4000, Liège, Belgium; d.baiwir@uliege.be (D.B.); m.fleron@uliege.be (M.F.)

[4] Cystic Fibrosis Reference Center, Cliniques Universitaires Saint-Luc, Université catholique de Louvain, B-1200, Brussels, Belgium; sophie.gohy@uclouvain.be (S.G.)

[5] Department of Pneumology, Cliniques Universitaires Saint-Luc, Université catholique de Louvain, B-1200, Brussels, Belgium; sophie.gohy@uclouvain.be (S.G.)

[6] Pole of Pneumology, ENT and Dermatology, Institut de Recherche Expérimentale et Clinique (IREC), Uni-versité Catholique de Louvain, B-1200, Brussels, Belgium; sophie.gohy@uclouvain.be (S.G.)

[a] corresponding authors

[b] equal contributors as last authors

Correspondence: bastien.burat@uliege.be (B.B.), gabriel.mazzucchelli@uliege.be (G.M.)

Phone: +32 (0) 4 366 34 11; fax: +32 (0) 4 366 43 87 (G.M.)

Supplementary Information

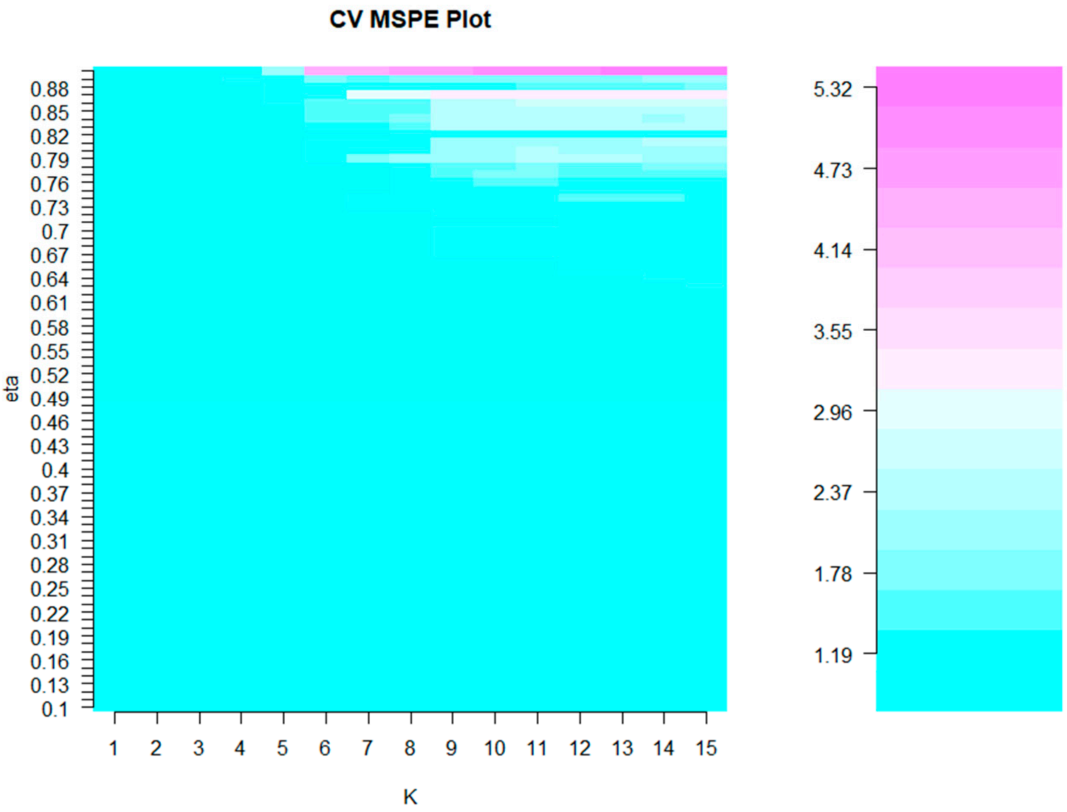

Supplementary Figure S1 – Mean Square Prediction Error (MSPE) plot for the determination of SPLS parameters. Minimal MSPE was obtained for η = 0.69 and k = 2.

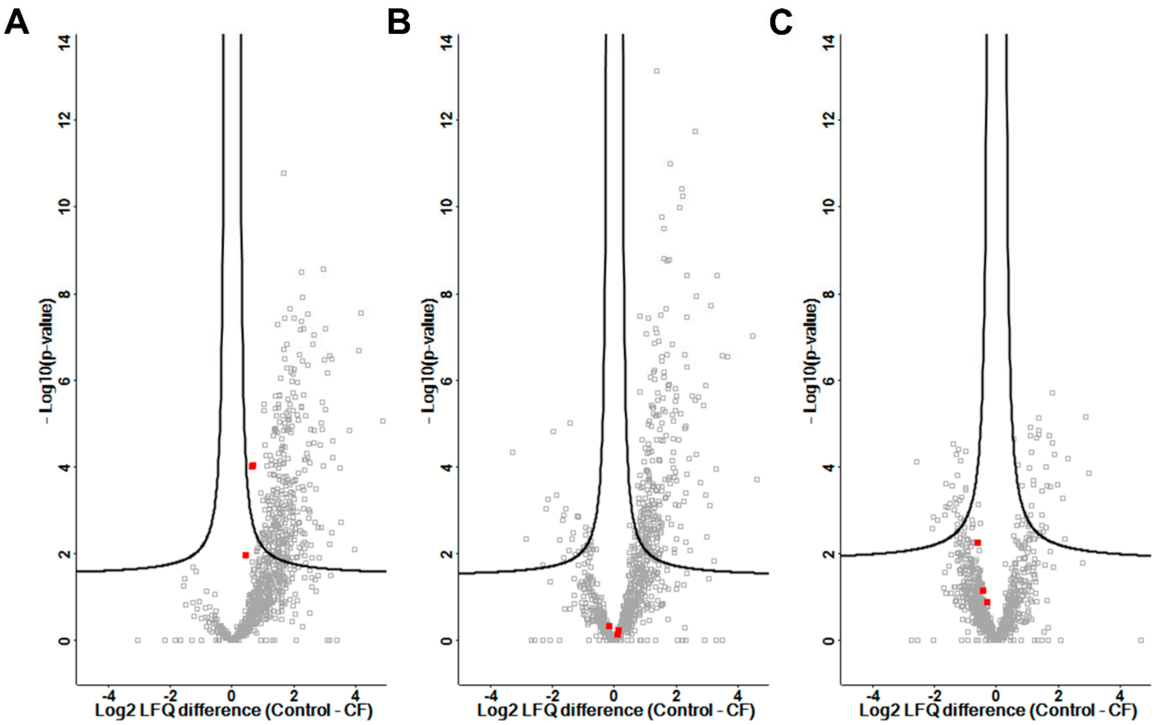

Supplementary Figure S2 – Separated LFQ normalization was applied to raw data since global LFQ normalization elicited a quantification bias. Volcano plot visualization after: A, no normalization, B, separated LFQ normalization, C, global LFQ normalization. Proteins from the MPDS Mix 1 standard mixture were highlighted in red filled squares.

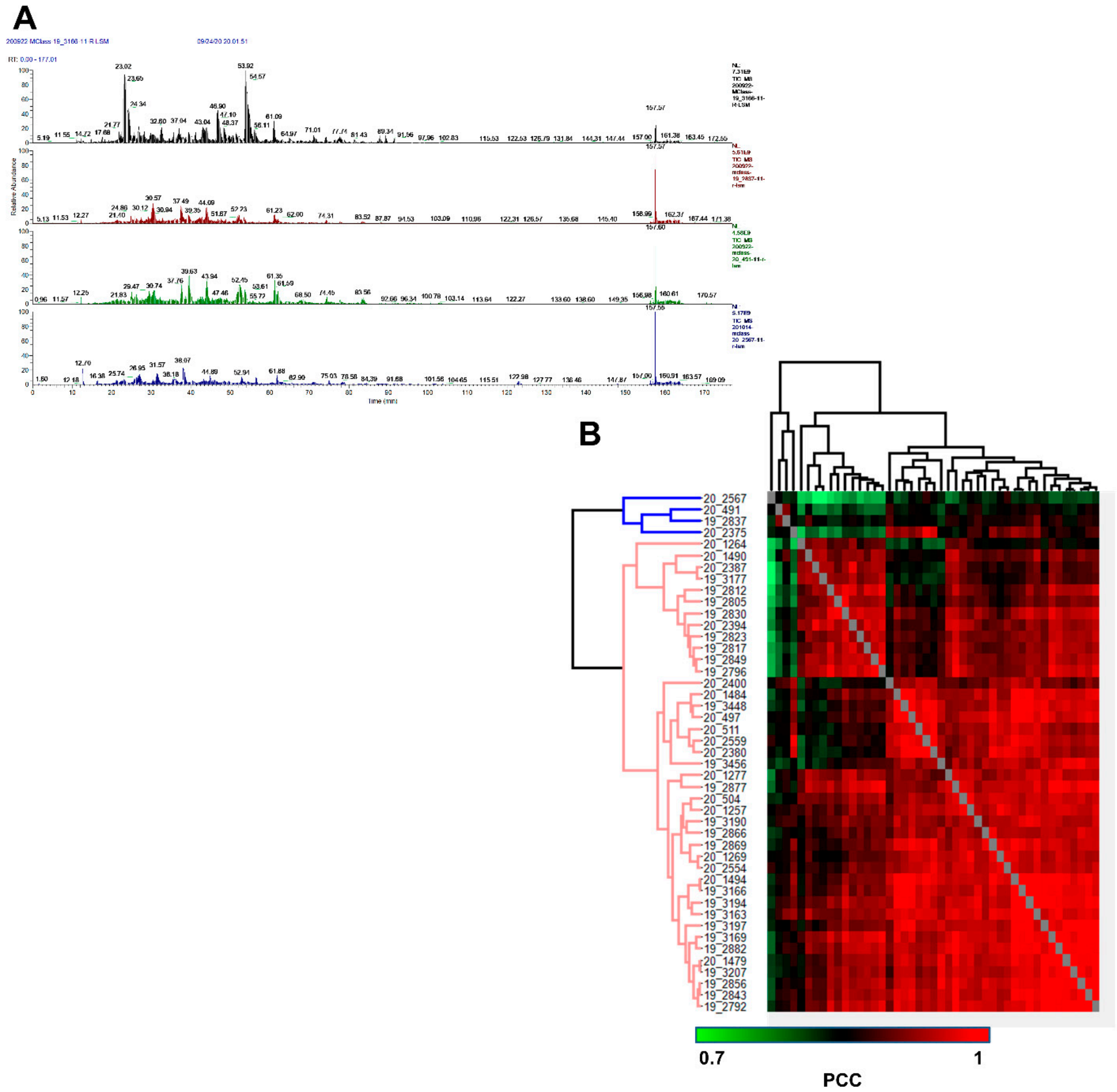

Supplementary Figure S3 – Discarded samples based on chromatogram discrepancy and poor correlation with other samples' protein profiles. a. TIC comparison. Reference TIC (black), TIC of discarded samples (red, green and blue). b. Heat-map representation of 30 control and 15 CF sweat protein profiles. Hierarchical clustering of Pearson's correlation coefficients generated using average Euclidian distance matrix.

Protein IDs	Age (years) (1)	Sex (2)	BMI (kg/m²) (3)	Collected volume (µL) (right arm) (4)	Collected volume (µL) (left arm) (5)	[Na+] (mM) (6)	[Cl-] (mM) (7)	[K+] (mM) (8)	[Protein] (µg/µL) (9)	Na+ (µmol) (10)	Cl- (µmol) (11)	K+ (µmol) (12)	Protein (µg) (13)
O00264	-0.006	0.009	-0.007	-0.003	-0.004	-0.022	-0.024	-0.012	0.0004	-0.02	-0.023	-0.013	-0.003
O14818	-0.006	0.007	-0.003	-0.001	-0.002	-0.02	-0.021	-0.012	-0.002	-0.017	-0.019	-0.011	-0.003
O15144	0.001	0.004	-0.008	-0.006	-0.005	0.001	0.001	0.005	0.007	-0.003	-0.002	-0.003	0.001
O43396	0.004	0.001	-0.006	-0.006	-0.004	0.01	0.01	0.01	0.008	0.005	0.006	0.002	0.002

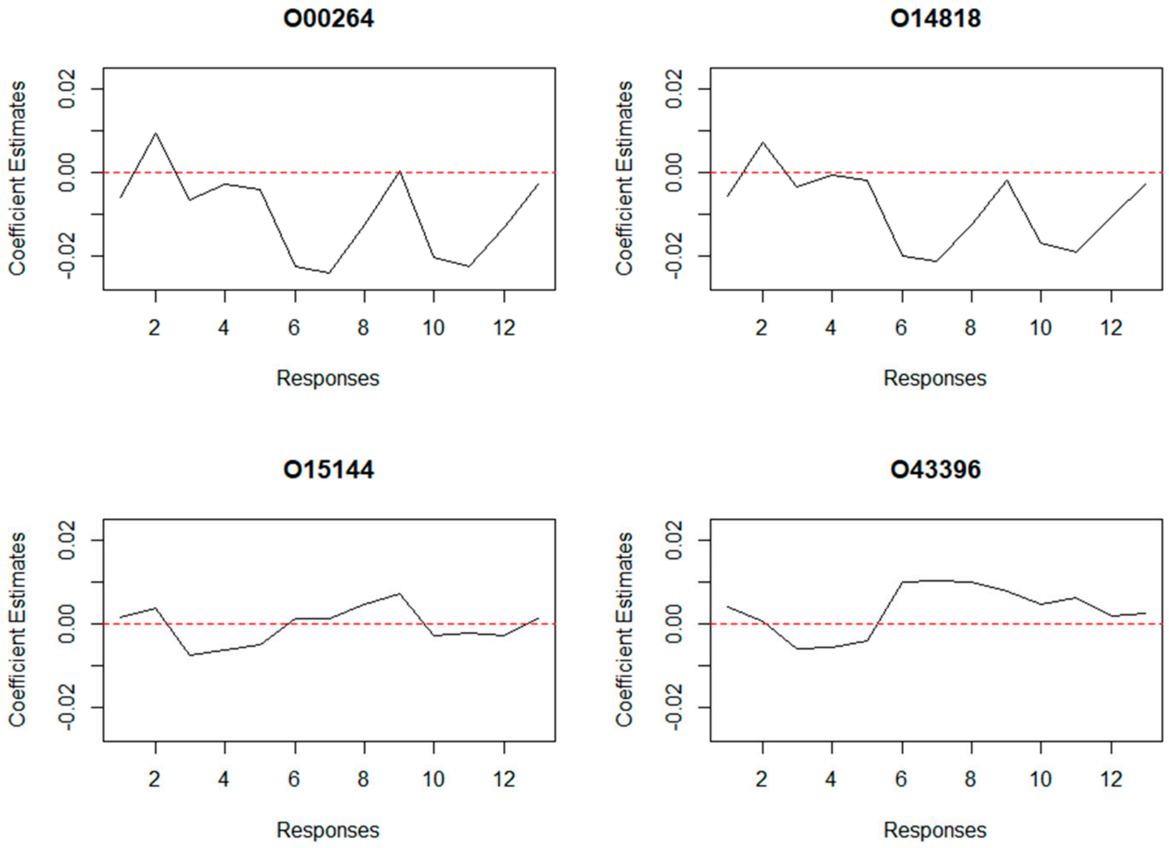

Supplementary Figure S4 – Plot of the estimated coefficients of the first four sweat proteins selected as important variables for all responses, following SPLS regression.

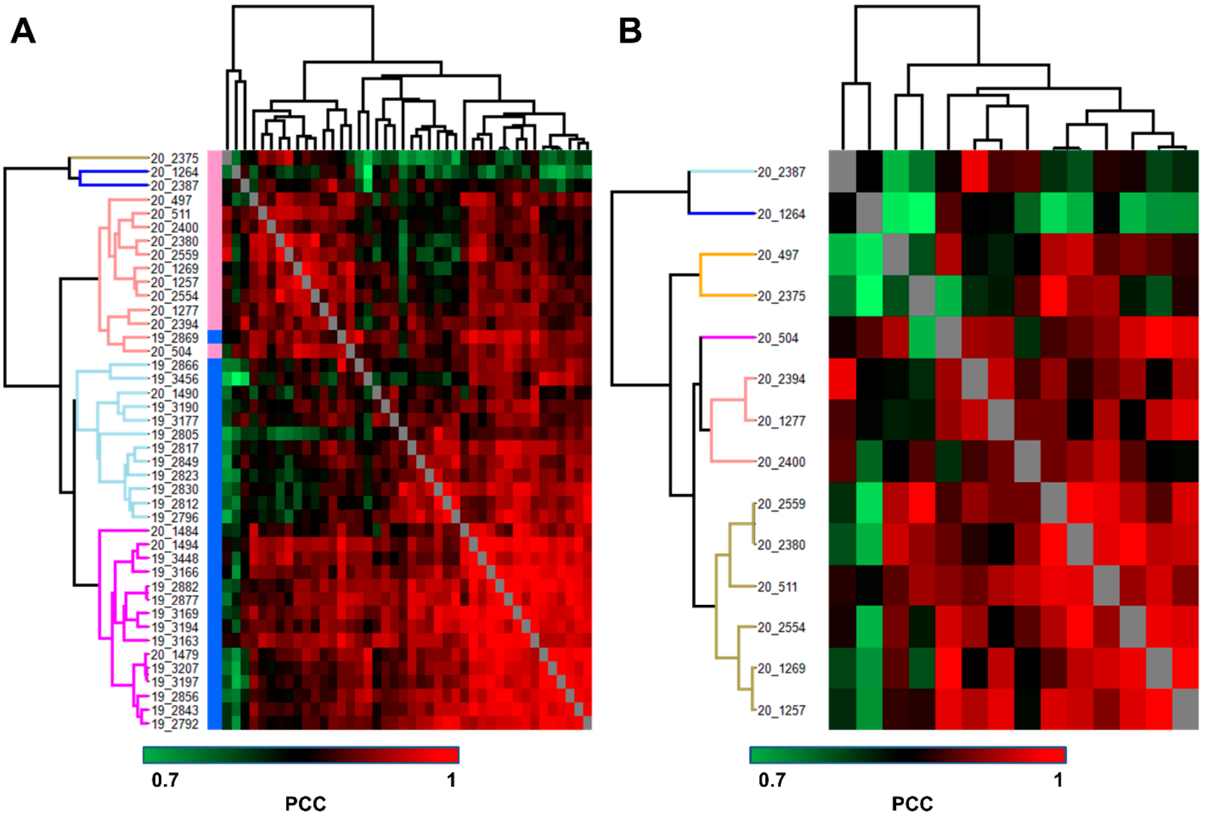

	PERMANOVA *p*-value
Age (years)	0.371
Sex	0.389
BMI (kg/m^2)	0.319
Volume (µL) (right)	0.174
Volume (µL) (left)	0.666
$[Na^+]$ (mM)	0.001 * * *
$[Cl^-]$ (mM)	0.001 * * *
$[K^+]$ (mM)	0.004 * *
[Protein] (µg/µL)	0.003 * *
Na^+ (µmol)	0.002 * *
Cl^- (µmol)	0.001 * * *
K^+ (µmol)	0.007 * *
Protein (µg)	0.325
CF	0.001 * * *

	PERMANOVA *p*-value
Age (years)	0.974
Sex	0.084
BMI (kg/m^2)	0.327
Volume (µL) (right)	0.932
Volume (µL) (left)	0.797
$[Na^+]$ (mM)	0.553
$[Cl^-]$ (mM)	0.893
$[K^+]$ (mM)	0.117
[Protein] (µg/µL)	0.067
Na^+ (µmol)	0.970
Cl^- (µmol)	0.934
K^+ (µmol)	0.421
Protein (µg)	0.233

Genotype	F508del: 0.026 *
	Allele A: 0.551
	Allele B: 0.306
	Genotype: 0.307
PI onset	0.215
Diabetes onset	0.77
Sputum microbiology	Colonization: 0.229
	PA infection: 0.125
	AF infection: 0.774
	A. sp. infection: 0.42
	SA infection: 0.926
	BCC infection: 0.516
	Co-infection : 0.398
FEV1 (L)	0.992
FEV1 %predicted	0.952

Supplementary Figure S5 – Sweat CF biomarker profiles discriminated patients with CF from control subjects, in correlation with CFTR genotype. A. Heat-map representation of control versus CF sweat CF biomarker profiles. Control samples (blue color bar), CF samples (pink color bar). B. Heat-map representation of CF sweat CF biomarker profiles. Hierarchical clustering of Pearson's correlation coefficients using average Euclidian distance matrix. PERMANOVA test for significance of correlation between clustering & clinical data distribution (number of permutations=999).
